# Real-world evidence of the effects of sodium-glucose co-transporter 2 inhibitors on the dosing of diuretics in patients with heart failure: a retrospective cohort study

**DOI:** 10.3389/fphar.2024.1366439

**Published:** 2024-04-02

**Authors:** Abdulaziz Alsalem, Mohammed M. Alsultan, Faisal Alqarni, Abdullah Almangour, Lolwa Alsharekh, Saleem Alenazi, Saleh Alzahrani, Raghad A. Almanqour, Abdullah Alazmi, Abdullah Alzahrani

**Affiliations:** ^1^ Department of Pharmacy, Security Forces Hospital, Riyadh, Saudi Arabia; ^2^ Department of Pharmacy Practice, College of Clinical Pharmacy, Imam Abdulrahman Bin Faisal University, Dammam, Saudi Arabia; ^3^ Department of Cardiology, Security Forces Hospital, Riyadh, Saudi Arabia; ^4^ College of Medicine Al-Jouf University, Sakaka, Saudi Arabia

**Keywords:** SGLT2 inhibitors, diuretics, heart failure, dapagliflozin, cardiovascular disease

## Abstract

**Background:** Heart failure (HF) was estimated to impact approximately 64 million individuals worldwide in 2017 and is predicted to rise in the coming years. Therefore, the aim of our study was to evaluate the effects of sodium-glucose transport protein 2 (SGLT2) inhibitors on the dosing of diuretics among individuals diagnosed with HF.

**Methods: **A retrospective cohort study was conducted at Security Forces Hospital in Riyadh, Saudi Arabia, between January 2018 and August 2022. The study included adult patients who were diagnosed with heart failure and received dapagliflozin and/or diuretic. A descriptive analysis was conducted to identify significant differences between both groups by using the chi-square test for categorical variables and the Student’s t*-*test for continuous variables. A logistic regression model was also run to identify the odds of each event. Statistical significance was indicated by *p* values less than .05.

**Results: **Overall reduction in diuretics was reported in 68 patients in the SGLT2 inhibitors plus diuretic therapy group, while in the diuretic therapy group 25 patients reported overall reduction in diuretics (OR = 4.81, 95% [2.74–8.45]). The reduction of the loop dose level was reported by 58 patients in the SGLT2 inhibitors plus diuretic group and by 25 patients in the diuretic group (OR = 3.48, 95% [1.98–6.11]). The discontinuation of thiazide was reported by 16 patients in the SGLT2 inhibitors plus diuretic therapy group, but by only two patients in the diuretic group (OR = 9.04, 95% [2.03–40.19]). After 6 months, ejection fraction was increased by 2.74 in the SGLT2 inhibitors plus diuretic group (*p* = .0019) and decreased by 2.56 in the diuretic group (*p* = .0485), both of which were statistically significant. The mean dose changes were decreased by 14.52 in the SGLT2 inhibitors plus diuretic group (*p* < .0001), which was statistically significant.

**Conclusion:** Treatment with SGLT2 inhibitors plus diuretic significantly reduced the patients’ diuretic requirements. Therefore, our finding supports the theoretical concept of minimizing the level of diuretic upon the initiation of SGLT2 inhibitors.

## 1 Introduction

Heart failure (HF) was estimated to impact approximately 64 million individuals worldwide in 2017 and is predicted to rise in the coming years (Lippi and Sanchis-Gomar, 2020). HF is a complex, potentially fatal illness categorized according to its significant economic burden, substantial morbidity, and mortality nationwide (Savarese et al., 2022). Standard care for HF has focused on several mechanisms, including the blocking of renin-angiotensin-aldosterone system and the sympathetic nervous system (Nishimura et al., 2017). Diuretic therapy (DT) is thought to be crucial for treating the symptoms of HF, and the guidelines strongly recommend the use of loop diuretics to relieve the symptoms of fluid overload (Nishimura et al., 2017; McDonagh et al., 2021; Heidenreich et al., 2022). The 2022 American College of Cardiology and the American Heart Association guidelines, as well as the 2021 European Society of Cardiology guidelines, both for the management of HF, recommend four medication classes that include sodium-glucose co-transporter 2 (SGLT2) inhibitors have emerged as promising agents in HF management, as a class 1 recommendation similar to beta-blockers, angiotensin-converting enzyme inhibitors (ACEIs), angiotensin receptor-neprilysin inhibitor (ARNIs), angiotensin receptor blockers (ARBs), and mineralocorticoid receptor antagonists (MRAs) for reduced ejection fraction in HF and class 2a recommendation for mildly reduced and preserved ejection fraction in HF (McDonagh et al., 2021; Heidenreich et al., 2022).

For patients diagnosed with both diabetes mellitus (DM) and heart failure with reduced ejection fraction (HFrEF) who are undergoing treatment with ACEIs or ARBs, the diuretic of choice is the thiazide or thiazide-like diuretic, such as hydrochlorothiazide or indapamide. If volume overload persists despite thiazide therapy, a loop diuretic such as furosemide can be introduced. In contrast, for those patients with DM and HF along with preserved ejection fraction (HFpEF) who are undergoing treatment with ACEIs or ARBs, the initial choice of diuretic is a loop diuretic such as furosemide. However, if adequate volume control is not achieved with a loop diuretic alone, a thiazide or thiazide-like diuretic can be introduced (Gérard et al., 2022).

The results of two major trials have contributed to SGLT2 inhibitors being strongly recommended by the guidelines. On the one hand, the Dapagliflozin and Prevention of Adverse Outcomes in Heart Failure (DAPA-HF) trial was carried out to assess the effectiveness of dapagliflozin in HF patients with lower ejection fraction, regardless of the presence or absence of diabetes.

Dapagliflozin is used in patients diagnosed with HF as part of their treatment regimen. It shown that participants who received dapagliflozin had a considerably lower chance of developing worsening HF or death from cardiovascular causes as compared to those who got a placebo (McMurray et al., 2019). On the other, the Cardiovascular and Renal Outcomes with Empagliflozin in HF was conducted to evaluate the efficacy of empagliflozin *versus* a placebo on top of guideline-directed medical therapy in patients with HF with reduced ejection fraction. The findings were similar to the findings of the DAPA-HF. Among patients who were receiving recommended therapy for HF, ones in the empagliflozin group had a lower risk of the composite of cardiovascular death and HF hospitalization regardless of their diabetes status (Packer et al., 2020). The initial sodium excretion with both bumetanide and dapagliflozin does not exhibit additive effects. However, weekly administration of one diuretic increases the initial sodium excretion that the other induces, suggesting a reciprocal adaptive natriuretic synergy (Wilcox et al., 2018).

The therapeutic effect of SGLT2 inhibitors in HF may derive from their diuretic effects and beneficial impact on renal physiology, both of which are attractive pathways to explore (Heerspink et al., 2016). By preventing the proximal tubule from absorbing glucose and sodium, SGLT2 inhibitors produce their diuretic effects (Becher et al., 2021). However, little is known about the effects of combining SGLT2 inhibitors with loop diuretic dosing. SGLT2 inhibitors operate by inhibiting the SGLT2 protein located in the renal proximal tubules. This mechanism decreases the reabsorption of filtered glucose, leading to increased urinary glucose excretion and consequently lowering blood glucose levels. Furthermore, SGLT2 inhibitors facilitate natriuresis and osmotic diuresis, resulting in a decrease in blood volume and systemic blood pressure. In patients diagnosed with both HF and type 2 DM, SGLT2 inhibitors offer various beneficial effects beyond glycemic management. Studies have demonstrated their capability to decrease the risk of cardiovascular events, including cardiovascular death, myocardial infarction, and stroke, in individuals with T2DM and established cardiovascular disease (Tang et al., 2022).

Dapagliflozin and metolazone were equally effective in decreasing congestion in patients with heart failure and resistance to loop diuretics. In contrast to those prescribed metolazone, those assigned to dapagliflozin experienced fewer biochemical abnormalities despite receiving a larger cumulative dose of furosemide (Yeoh et al., 2023).

The RECEDE-CHF trial assessed patients with type 2 diabetes and HF with reduced ejection fraction using SGLT2 inhibitors in combination with diuretics. Participants were randomly assigned to receive either empagliflozin 25 mg or a placebo once daily for 6 weeks. The trial concluded that empagliflozin causes a remarkable increase in 24-h urine volume without an increase in urinary sodium when used alongside a loop diuretic compared with the placebo (Mordi et al., 2020). Another randomized controlled trial, aimed at assessing the role of SGLT2 inhibitors in acute decompensated HF (ADHF), involved the comparison of empagliflozin 25 mg daily against a placebo and loop diuretics. The trial showed that the addition of empagliflozin daily to standard medical treatment with ADHF resulted in a 25% significant elevation in cumulative urine output over 5 days compared with the placebo (Schulze et al., 2022). Evidence from both clinical trials and real-world clinical practice unequivocally confirms that SGLT2 inhibitors are deemed “kidney safe” and do not predispose individuals to acute kidney injury (AKI). This conclusion is reinforced by data from numerous cardiovascular outcome trials, particularly emphasizing findings from the CREDENCE trial. These investigations have demonstrated that SGLT2 inhibitors offer protection against the progression of diabetic kidney disease, representing a significant advancement in its treatment. This milestone is noteworthy for both the nephrology community and patients with diabetes-related kidney and/or cardiovascular diseases, as SGLT2 inhibitors emerge as the first novel treatment option in the last 2 decades to effectively combat the progression of diabetic kidney disease. Moreover, beyond their efficacy in treating diabetic kidney disease, it is now established that SGLT2 inhibitors do not elevate the risk of AKI, providing reassurance regarding their safety profile in this aspect (Sridhar et al., 2020).

A significant decrease in the likelihood of experiencing AKI with the administration of SGLT2 inhibitors, evidenced by a 36% decrease in AKI odds across RCTs. Moreover, the analysis underscores similar benefits among different SGLT2 inhibitors agents in moderating serious AE and AE rates. Nevertheless, a concomitant increase in the reporting of hypovolemia-related AEs prompts a deeper exploration of the interplay between SGLT2 inhibitors therapy, renal outcomes, and overall treatment approaches (Menne et al., 2019).

A retrospective cohort observational study was conducted among patients with diabetes to assess the safety of SGLT2 inhibitors combined with loop diuretics compared with SGLT2 inhibitors alone. In that study, 98 patients were given SGLT2 inhibitors plus loop diuretics while 302 patients were given SGLT2 inhibitors alone. A month later, the combined use of SGLT2 inhibitors and loop diuretics was found to be associated with a significant increase in volume-depletion events after 12 months of DT (Rahhal et al., 2022). Based on the conflicting results in the mentioned studies and because clinical outcomes regarding the effects of SGLT2 inhibitors on the dosing of diuretic medications have not been widely evaluated, especially not in Saudi Arabia, the aim of our study was to evaluate the effects of SGLT2 inhibitors on the dosing of diuretics among individuals diagnosed with HF.

## 2 Methods

### 2.1 Setting and design

Our study was a single-center, retrospective cohort study with an observational design conducted at the Security Forces Hospital in Riyadh, Saudi Arabia. We targeted patients with HF with reduced ejection fraction or HF with preserved ejection fraction who were receiving SGLT2 inhibitors and loop diuretics from January 2018 until August 2022.

### 2.2 Population

The study population was all patients at the Security Forces Hospital in Riyadh diagnosed with HF in the selected period (i.e., heart failure patients either with reduced ejection fraction or preserved ejection fraction regardless of the presence of diabetes). The study cohort was divided into two groups: patients on loop diuretics with SGLT2 inhibitors (dapagliflozin) added later and patients on loop diuretics only.

### 2.3 Primary and secondary outcomes

The primary outcome was changes in the need for DT after the addition of dapagliflozin when compared with treatment without the addition of dapagliflozin. Secondary outcomes included a change in the ejection fraction and systolic blood pressure as well as the worsening of renal function (i.e., increased serum creatinine (SCr) by >30%).

### 2.4 De-escalation of DT

We defined the reduction of the patient’s needs for DT as either the discontinuation of thiazide therapy after the initiation of dapagliflozin or a reduction in loop diuretic dose after the initiation of dapagliflozin.

### 2.5 Inclusion and exclusion criteria

All patients with HF who received loop diuretics were included. All patients who were not on loop diuretics, had chronic kidney disease with a creatinine clearance of less than 30 mL/min/1.73 m^2^, or had acute kidney injury defined as rise in serum creatinine by at least twice the baseline following the Kidney Disease Improving Global Outcomes criteria (Yeoh et al., 2023) and using an SGLT2 inhibitors for less than 3 months were excluded.

### 2.6 Data collection and variables

From January 2018 until August 2022, all relevant patients’ medical data were collected from the hospital’s electronic medical records, and a retrospective chart review was conducted. The information was gathered on a predetermined data collection sheet, on which the patient’s age, weight, body mass index (BMI) sex, systolic blood pressure (SBP), left ventricular ejection fraction (LVEF), serum creatinine levels, comorbidities (hypertension, diabetes mellitus, chronic kidney disease, chronic artery disease), and medications use (Beta blockers (BB), Aldosterone Antagonist (AA), ACEIs, ARBs, angiotensin receptor/neprilysin inhibitors (ARNIs) were recorded, along with, the SGLT2 inhibitors dose, and loop diuretics dose.

### 2.7 Data analysis

A descriptive analysis was conducted to identify significant differences between both groups by using the chi-square test for categorical variables and the Student’s t*-*test for continuous variables. Percentages and frequencies were used for the categorical variables, while means and standard deviations were used for the continuous variables. An unadjusted logistic regression model was run to identify the odds of each event. We checked normality by using the Shapiro–Wilk test and the Wilcoxon signed-rank test. Statistical significance was indicated by *p* values less than .05. All statistical analyses were performed using SAS V.9.4.

## 3 Results

Table 1 shows the baseline characteristics of the patients. The mean age (*SD*) of patients in the SGLT2 inhibitors plus DT group was 59.81 (13.62) and of patients in the DT group was 62.81 (14.20), which was not statistically significant (*p* = .06). Seventy-six patients (30.65%) in the SGLT2 inhibitors + DT group were male compared with 75 patients (30.24%) in the DT group (*p* = 1.00); by contrast, 48 patients (19.35%) in the SGLT2I + DT group were female compared with 49 patients (19.76%) in the DT group. The mean (*SD*) systolic blood pressure (mm Hg) was 127.72 (21.55) in the SGLT2 inhibitors + DT group and 126.08 (21.91) in the DT group (*p* = .55). The mean (*SD*) serum creatinine level (umol/L) was 91.30 (44.45) in the SGLT2 inhibitors + DT group and 129.08 (128.45) in the DT group (*p = .*002). The mean baseline left ventricular ejection fraction (*SD*) was 37.69 (14.03) in the SGLT2 inhibitors + DT group and 47.80 (15.35) in the DT group (*p < .*001). The mean (*SD*) weight (kg) was 84.14 (22.2) and 82.26 (19.4) in the SGLT2 inhibitors + DT group and DT group, respectively (*p = .*4792), while the mean (*SD*) height (cm) was 161.70 (12.96) and 162.26 (10.08) in the respective groups (*p = .*7079). The BMI mean (*SD*) was 31.98 (8.82) and 31.13 (7.10) in the SGLT2 inhibitors + DT group and the DT groups, also respectively (*p = .*4003). Regarding comorbidities, most participants in both groups had hypertension (SGLT2 inhibitors + DT group: *n =* 98, 39.52%; DT group: *n =* 107, 43.15%; *p = .*1792). Most participants had diabetes (SGLT2 inhibitors + DT group: *n =* 102, 41.13%; DT group: *n =* 88, 35.48%; *p = .*0506), and most did not suffer from Chronic Kidney Disease (SGLT2 inhibitors + DT group: *n =* 99, 39.92%; DT group: *n =* 92, 37.10%; *p = .*3653). However, most patients in both groups did have Chronic Artery Disease (SGLT2 inhibitors + DT group: *n =* 82, 33.06%; DT group: *n =* 76, 30.65%; *p = .*5092). Regarding medication use, BB users totaled 116 (46.77%) and 110 (44.35%) in the SGLT2 inhibitors + DT group and DT group, respectively (*p = .*2639), whereas AA users totaled only 45 (18.15%) and 21 (8.47%) in the SGLT2 inhibitors + DT group and DT group, respectively (*p = .*0009). Last, in the SGLT2 inhibitors + DT group and DT group, ACEI users totaled 64 (25.81%) and 55 (22.18%; *p = .*0041), ARB users totaled 29 (11.69%) and 35 (14.11%; *p = .*0041), and ARNI users totaled 27 (10.89%) and 16 (6.45%; *p = .*0041), all respectively.

**TABLE 1:** Baseline characteristics of the HF patients.

Characteristic	SGLT2I plus diuretics (*N* = 124)	Diuretics (*N* = 124)	*p* -value*
	*n* (%)	*n* (%)	
Age, in years—Mean (*SD*)	59.81 (13.62)	62.81 (14.20)	.0615
Mean weight, in kg- Mean (*SD*)	84.14 (22.2)	82.26 (19.4)	.4792
BMI—Mean (*SD*)	31.98 (8.82)	31.13 (7.10)	.4003
Sex			
Male	76 (30.65%)	75 (30.24%)	1.00
Female	48 (19.35%)	49 (19.76%)	
Systolic blood pressure, in mm Hg–Mean (*SD*)	127.72 (21.55)	126.08 (21.91)	.5536
Serum creatinine, umol/L–Mean (*SD*)	91.30 (44.45)	129.08 (128.45)	.0022
Left ventricular ejection fraction–Mean (*SD*)	37.69 (14.03)	47.80 (15.35)	<.0001
Comorbidities			
Hypertension			
Yes	98 (39.52%)	107 (43.15%)	.1792
No	26 (10.48%)	17 (6.85%)	
Diabetes Mellitus			
Yes	102 (41.13%)	88 (35.48%)	.0506
No	22 (8.87%)	36 (14.52%)	
Chronic Kidney Disease			
Yes	25 (10.08%)	32 (12.90%)	.3653
No	99 (39.92%)	92 (37.10%)	
Chronic Artery Disease			
Yes	82 (33.06%)	76 (30.65%)	.5092
No	42 (16.94%)	48 (19.35%)	
Medication use			
Beta blockers			
Yes	116 (46.77%)	110 (44.35%)	.2639
No	8 (3.23%)	14 (5.65%)	
Aldosterone Antagonist			
Yes	45 (18.15%)	21 (8.47%)	.0009
No	79 (31.85%)	103 (41.53%)	
ACEIs, ARBs, or ARNIs			
ACEIs	64 (25.81%)	55 (22.18%)	.0041
ARBs	29 (11.69%)	35 (14.11%)	
ARNIs	27 (10.89%)	16 (6.45%)	
None	4 (1.61%)	18 (7.26%)	

*p-value <.05.

Table 2 shows the results of the logistic regression analysis comparing the two groups (i.e., SGLT2 inhibitors + DT vs. DT). Overall reduction was reported in 68 patients in the SGLT2 inhibitors + DT group, while in the DT group 25 patients reported overall reduction in diuretics (OR = 4.81, 95% CI [(2.74–8.45)]). The reduction of the loop dose level was reported by 58 patients in the SGLT2 inhibitors + DT group and by 25 in the DT group (OR = 3.48, 95% CI [(1.98–6.11)]). The discontinuation of thiazide was reported by 16 patients in the SGLT2 inhibitors + DT group but by only two patients in the DT group (OR = 9.04, 95% CI [(2.03–40.19)]). Moreover, worsening renal function was observed in 29 patients in the SGLT2 inhibitors + DT group *versus* 26 patients in the DT group (OR = 1.15, 95% CI [(0.63–2.10)]).

**TABLE 2:** Logistic regression of HF Patients.

Event	SGLT2I plus diuretics (N)	Diuretics (*N*)	Odds ratio (95%)*
Overall reduction in diuretics	68	25	4.81 (2.74–8.45)
Reduction of loop Dose level	58	25	3.48 (1.98–6.11)
Discontinuation of thiazide	16	2	9.04 (2.03–40.19)
Worsening renal function (i.e., increased SCr by >30%)	29	26	1.15 (0.63–2.10)

*SGLT2I plus diuretics *versus* diuretics only.

Table 3 shows the mean changes from baseline to after 6 months. After 6 months, Ejection fraction (EF) was 2.74 in the SGLT2 inhibitors + DT group (*p = .*0019) and −2.56 in the DT group (*p = .*0485), which was statistically significant. The mean dose change was −14.52 in the SGLT2 inhibitors + DT group (*p* < .0001), which was also statistically significant; however, in the DT group, the mean dose change was 2.27 (*p = .*2232), which was statistically non-significant. Moreover, significant changes were found in Scr in the SGLT2 inhibitors + DT group (9.89; *p = .*0002) and in the DT group (12.23; *p < .*001). Last, non-significant changes were noted in mean SBP in the SGLT2 inhibitors + DT group (−1.15; *p = .*4473) and in the DT group (3.25; *p = .*1594).

**TABLE 3:** Mean changes from baseline to 6 months in HF Patients.

	SGLT2I plus diuretics therapy	*p-*value*	Diuretic therapy only	*p*-value*
Ejection fraction	2.74	.0019	−2.56	.0485
Dose of loop diuretics	−14.52	<.0001	2.27	.2232
Systolic blood pressure	−1.15	.4473	3.25	.1594
Serum creatinine	9.89	.0002	12.23	<.0001

*p-value <.05.

## 4 Discussion

Our findings showed that therapy with SGLT2 inhibitors plus diuretic significantly reduced the patients’ diuretic requirements. After 6 months of EF and Scr in the SGLT2 inhibitors plus DT, there was a statistically significant increase in the mean change. Additionally, when the patients started using SGLT2 inhibitors 6 months later, the dose of loop diuretics was dramatically reduced.

The SGLT2 inhibitors lower cardiovascular risk and prevent diabetic kidney damage, they are a desirable treatment choice for type 2 diabetes (Zannad et al., 2020). Nevertheless, it is crucial to recognize the constraints of our research, including potential biases inherent in its retrospective design and the possibility of incomplete or inaccurate documentation in medical records. Further discussion on the shortcomings of the control group and additional background information regarding their characteristics could enhance the interpretation of our results. Despite the absence of diabetes, those agents are recommended for patients with HF (Maddox et al., 2021). The benefits of SGLT2 inhibitors therapy in HF can be attributed to multiple mechanisms, including the induction of an osmotic diuretic and natriuretic effect, which may reduce the severity of HF exacerbations (Lam et al., 2019). Those medications are usually prescribed for patients with HF in addition to other medications that induce diuresis, including loop diuretics and guideline-directed medical therapy. However, currently little data indicate whether similar effects on diuretic de-escalation are evident in the real-world use of SGLT2 inhibitors in those patients with HF. SGLT2 inhibitors have gained recognition for their efficacy in managing various conditions beyond diabetes, including heart failure and chronic kidney disease.

Our study included 248 patients with HF. We defined the reduction of the patient’s need for DT as either the discontinuation of thiazide therapy after the initiation of dapagliflozin or the reduction in loop diuretics after the initiation of dapagliflozin. The overall reduction in diuretics was reported in 68 patients (54.8%) in the SGLT2 inhibitors + DT group but in only 25 patients (20.16%) in the DT group (20.16%). Regarding loop diuretics, the dose reduction was reported in 57 patients (46.7%) in the SGLT2 inhibitors + DT group and in 25 patients (20.16%) in the DT group. Regarding thiazide diuretics, the discontinuation was reported by 16 patients (12.9%) in the SGLT2 inhibitors + DT group but by only two patients (1.6%) in the DT group.

According to a study in the literature, approximately 8% of patients experienced a reduction in the dose of loop diuretics 6 months after SGLT2 inhibitors were commenced which is in line with our results (Trudeau et al., 2023). However, our findings were inconsistent to the results of a published analysis of the DAPA-HF trial showing that, after the addition of dapagliflozin to DT, most patients did not experience a change in the dose of diuretics during follow-up, and the mean daily dose of diuretics did not change. However, that analysis examined changes in doses at specified time points, and fluctuations in doses between those points were not accounted for (Grodin and Tang, 2020). In our study, the worsening in renal function was observed in 29 patients (23.3%) in the SGLT2 inhibitors + DT group *versus* 26 patients (20.9%) in the DT group, which was non-significant. By contrast, a study demonstrated that SGLT2 inhibitors initially reduced glomerular filtration rate compared with the placebo group in the long term and this discovery underscores the substantial advantages of dapagliflozin for individuals with chronic kidney disease, regardless of their diabetic status. It emphasizes a noteworthy decrease in the likelihood of encountering a combined outcome, which includes a significant decrease in estimated glomerular filtration rate (GFR), progression to end-stage kidney disease, or mortality attributed to renal or cardiovascular causes (Heerspink et al., 2020). However, our findings are consistent with the findings of the DAPA-HF trial, which revealed that the incidence of the worsening of renal function was not significant between the dapagliflozin group and the placebo group (McMurray et al., 2019).

After 6 months from the baseline, we found that the ejection fraction had increased by 2.74 in the SGLT2 inhibitors + DT group (*p = .*0019) and decreased by −2.56 in the DT group (*p = .*0485), which was statistically significant. That finding was expected and consistent with the results of landmark SGLT2 inhibitors trials (McMurray et al., 2019; Packer et al., 2020). Concerning blood pressure, mean changes in SBP from baseline to 6 months were not significantly changed. That finding conflicts with the results of the DAPA-HF trial, which showed a significant reduction in SBP between the dapagliflozin group compared with the placebo group (McMurray et al., 2019).

The SGLT2 inhibitors + DT group experienced a statistically significant reduction of −14.52 in mean dose change from baseline (*p* < .0001), which was statistically significant. In the DT group, however, the mean dose change rose by 2.27 (*p = .*2232), which was statistically non-significant. In a published study they assessed the effects of dapagliflozin *versus* a placebo in three subgroups: no diuretics, non-loop diuretics, and loop diuretics (i.e., furosemide) with equivalent doses of <40, 40, and >40 mg, respectively. Of 6,263 randomly selected patients, 683 (10.9%) were not taking any diuretics, 769 (12.3%) were taking a non-loop diuretic, and 4,811 (76.8%) were taking a loop diuretic at baseline. The benefits of dapagliflozin treatment on the primary composite outcome were consistent regardless of the diuretic use category (*p*_interaction_ = .64) or loop diuretic dose (*p*_interaction_ = .57). The new initiation of loop diuretics was reduced by 32% due to dapagliflozin but had no impact on discontinuations or disruptions during follow-up. The placebo arm of the study exhibited a longitudinal increase in the mean dose of loop diuretics, which was significantly reduced with dapagliflozin treatment (Chatur et al., 2023). As found in our study, using dapagliflozin prompted a significant decrease in the need for new loop diuretics over time.

When SGLT2 inhibitors and MRA are used together, they have a diuretic effect, which poses a concern for safety, including renal dysfunction. However, a recent analysis of the DAPA-HF showed that dapagliflozin was similarly efficacious and safe in patients with Heart failure with reduced ejection fraction (HFrEF) taking or not taking an MRA, which supports the combined use of the drugs (Shen et al., 2021).

Our study is the first conducted in Saudi Arabia to assess the impact of SGLT2 inhibitors on the dosing of diuretic medication in patients with HF. Furthermore, our study will highlight the need to improve medical aspects and give healthcare practitioners insight into some of the challenges associated with providing treatment to HF patients in Saudi Arabia. However, our study had some limitations. First, it was a single-center study. Second, there was potential for incomplete, inaccurate, or missing documentation in patient medical records due to the study’s retrospective design. Despite those limitations, our significant findings support the theoretical concept of minimizing the needed level of DT upon the initiation of SGLT2 inhibitors.

## 5 Conclusion

In conclusion, our study indicates that 54.8% of HF patients treated with SGLT2 inhibitors in addition to DT had a reduction in DT. Treatment with SGLT2 inhibitors plus DT significantly reduced their requirements for diuretics. That finding supports the theoretical concept of minimizing the level of DT needed upon the initiation of SGLT2 inhibitors. However, a large-scale prospective study on patients with HF treated with SGLT2 inhibitors plus DT is needed in the future.

## Data Availability

The raw data supporting the conclusion of this article will be made available by the authors, without undue reservation.
